# Maternal exposure to O_3_ and NO_2_ may increase the risk of newborn congenital hypothyroidism: a national data-based analysis in China

**DOI:** 10.1007/s11356-021-13083-6

**Published:** 2021-03-02

**Authors:** Cuifang Qi, Li Shang, Wenfang Yang, Liyan Huang, Liren Yang, Juan Xin, Shanshan Wang, Jie Yue, Lingxia Zeng, Mei Chun Chung

**Affiliations:** 1 Department of Obstetrics and Gynecology, Maternal & Child Health Center, The First Affiliated Hospital of Xi’an Jiaotong University, No. 277, West Yanta Road, Xi’an,, Shaanxi 710061 People’s Republic of China; 2 School of Public Health, Xi’an Jiaotong University Health Science Center, Xi’an,, Shaanxi 710061 People’s Republic of China; 3 Department of Public Health and Community Medicine, Tufts University School of Medicine, Boston, MA USA

**Keywords:** Air pollution, Congenital hypothyroidism, Maternal, Newborn, Cutoff, China

## Abstract

Maternal exposure to air pollution during pregnancy is associated with adverse outcomes in the offspring, but limited studies focused on the impacts of gaseous air pollution on newborn congenital hypothyroidism (CH). Therefore, a national data-based analysis was conducted to explore the association between maternal exposure to gaseous air pollution and the incidence of CH in China. Annual average exposure levels of SO_2_, NO_2_, CO, and O_3_ from January 1, 2014, to December 30, 2014, were acquired from the Chinese Air Quality Online Monitoring and Analysis Platform. The annual incidence of newborn CH from October 1, 2014, to September 30, 2015, was collected from the Chinese Maternal and Child Health Surveillance Network. Temperature and toxic metal in wastewater in 2014 were also collected as covariates. Maternal exposure to O_3_ and NO_2_ in 1 μg/m^3^ level increment was positively associated with newborn CH, with an OR of 1.055 (95% CI 1.011, 1.102) and 1.097 (95% CI 1.019, 1.182) after adjusting for covariates completely. Compared with the lowest level of O_3_, maternal exposure to the 4th quartile of O_3_ was positively associated with newborn CH (OR 1.393, 95% CI 1.081, 1.794) after adjusting for covariates completely. And the 3rd and 4th quartiles of NO_2_ were associated positively with CH (OR 1.576, 95% CI 1.025, 2.424, and OR 1.553, 95% CI 0.999, 2.414, respectively) compared with the lowest level of NO_2_. By fitting the ROC curve, 93.688 μg/m^3^ in O_3_ might be used as cutoff to predict the incidence of newborn CH in China.

**Supplementary Information:**

The online version contains supplementary material available at 10.1007/s11356-021-13083-6.

## Introduction

Atmospheric pollution has become a global environmental burden and caused widespread public concern (Gao et al. 2016). In China, the deterioration of air quality has become a serious issue because of the rapid development of urbanization construction and industrialization in recent years. At the end of the last century, there were 7 cities in China among 10 cities with the most serious air pollution worldwide (Chay and Greenstone 2003). And there were only 16 cities that met the air quality standard among 161 tested cities according to the Bulletin of the Environment State issued by the Chinese Environmental Protection Ministry in 2014. The exposure level of air pollution in China was significantly higher than those in Europe and America (Donkelaar et al. 2010). The Chinese air quality situation was not optimistic.

Epidemiological studies have confirmed that air pollution is directly or indirectly related to the residents’ health and global diseases (Nascimento carvalho et al. 2010; Shaddick et al. 2018; Wang et al. 2017), such as respiratory and cardiovascular disease (Yang et al. 2019). Pregnancy is critical for the whole life process; multiple factors including maternal exposure to air pollution during pregnancy could cause adverse outcomes in pregnant women and infants. Maternal exposure to air pollution such as nitrogen oxides (NO_*x*_), sulfur dioxide (SO_2_), ozone (O_3_), and particulate matter (PM) before or during pregnancy was associated with maternal complications such as gestational diabetes mellitus (GDM) and gestational hypertension disease (Fleisch et al. 2014; Hooven et al. 2011; Malmqvist et al. 2013; Robledo et al. 2015; Wang et al. 2013; Wu et al. 2011), and it was found to be consistently associated with abortion and stillbirth (Dastoorpoor et al. 2018; Yang et al. 2018). For offspring, maternal exposure to air pollution might cause small for gestational age (SGA), preterm birth (PTB), coarctation of the aorta, tetralogy of Fallot, and other congenital anomalies (Ballester et al. 2010; Nieuwenhuijsen et al. 2013; Song et al. 2017; Vrijheid et al. 2011). Disease and economic afford from adverse pregnancy outcomes caused by air pollution was enormous which should be valued. Until now, researches on air pollution during pregnancy were mainly focused on maternal complications, fetal development, and congenital malformation; limited studies paid attention to metabolic diseases such as newborn congenital hypothyroidism (CH) (Li et al. 2019).

Thyroid function is critical for regulating body growth and metabolism; it plays an important role in neurodevelopment. During pregnancy, a subtle change in maternal thyroid function can influence fetal growth even causing cognitive deficits during childhood, finally damaging mental and physical development in humans the future (Jansen et al. 2019; Korevaar et al. 2016; Seo et al. 2017). Researches verified that maternal and fetal thyroid function was susceptible to prenatal exposure to PM and NO_2_, especially in early pregnancy and mid-pregnancy (Howe et al. 2018; Zhao et al. 2019). The prenatal exposure to air pollution could change the concentration of the thyroid-stimulating hormone (TSH) and the ratio of thyroxine (T4)/triiodothyronine (T3) in cord blood (Janssen et al. 2017). Furthermore, low birth weight (LBW) and GDM might be associated with thyroid disease. PTB also has a significant influence on newborn CH. LBW, PTB, and GDM were confirmed to be associated with maternal exposure to air pollution, which may affect the occurrence of newborn CH indirectly (David et al. 2018).

Therefore, we wondered whether air pollution could affect the incidence of newborn CH. And it is necessary to explore the influential association between maternal exposure to air pollution and the occurrence of newborn CH. The worldwide incidence of CH ranges from 1 per 2000 to 1 per 4000 live births; western countries suggest an occurrence of CH of about 1 per 3000–4000. In China, it approximately was 1 per 2000–2500 live births, even reaching 1 per 1089 in Fujian province (Mansoor 2020; Zhou et al. 2020). The incidence of CH in China was obviously higher than most of countries (Fu et al. 2017; Sun et al. 2018). China is still a developing country; air pollution is a serious problem and will exist persistently in the future decades. Currently, researches on air pollution during pregnancy were mainly focused on PM, rather than gaseous air pollutants including SO_2_, NO_2_, carbon monoxide (CO), and O_3_. Therefore, we are going to explore the associations between maternal exposure to gaseous air pollutants and the newborn incidence of CH. We hope to calculate the cutoff value of air pollutants, and it may be helpful to set an interim goal in air pollution control, and to reduce the incidence of newborn CH in China.

## Materials and methods

### Study design

A national data-based analysis was conducted to explore the associations between maternal exposure to gaseous air pollutants during pregnancy and the incidence of newborn CH of 30 provinces in China. Annual exposure levels of SO_2_, NO_2_, CO, and O_3_ were collected from January 1, 2014, to December 30, 2014, considering that the gestation period lasts about 10 months. There was a lag period to observe the influence of air pollution on the CH in the newborn, so the annual incidence of newborn CH was collected from October 1, 2014, to September 30, 2015.

### Exposure assessment

The monthly average exposure levels of SO_2_, NO_2_, O_3_, and CO of 367 major cities in China from January 1, 2014, to December 30, 2015, were collected in the study. The 367 cities were representative of the major prefecture-level cities of 30 provinces in China (the data of air pollution in Tibet, Taiwan, Hong Kong, and Macau were not included). The monthly average levels of air pollution in each city were provided by the Chinese Air Quality Online Monitoring and Analysis Platform (https://www.aqistudy.cn/). The data of gaseous air pollution in this platform originated from the real-time monitoring data, which were recorded by the Ministry of Ecology and Environment of the People’s Republic of China. The concentration of each pollutant was continuously measured by the monitors in 367 cities of China.

### Incidence of CH

The annual incidence of newborn CH from October 1, 2014, to September 30, 2015, in 30 provinces of China derived from the Annals on the Chinese Neonatal Metabolic Disease Screening in 2015. The annals were published in Chinese Maternal and Child Health Surveillance Network (https://www.mchscn.org/) in February 2017, compiled by the National Office of the Maternal and Child Health Surveillance (NOMCHS), based on the Chinese Newborn Screening Information System (CNSIS). CNSIS is responsible for the newborn screening program mainly for mentalism disease and birth defects across China, including the screening of CH. Whole blood was collected from every newborn on filter papers to measure the serum levels of thyroid-stimulating hormone (TSH) between 72 h and 7 days following birth. Blood sample collection was postponed to 20 days following birth for the PTB, LBW, or sick neonates. Once the concentration of TSH was higher than 10–20 μIU/ml in double testing, the cases were required to be followed up and subjected to further diagnosis by determination of the thyroid hormone. The diagnosis of newborn CH was confirmed by pediatric endocrinologists who accepted the professional training based on serum thyroid function-related hormone. Each newborn diagnosed with CH was reported to a local newborn screening center (LNBSC). Subsequently, a provincial newborn screening center (PNBSC) was established to guide and supervise neonatal screening practices in LNBSC. The screening results were reported by PNBSC and published by NOMCHS annually. The screening and diagnosis of CH need to follow the Technological Guideline on National Newborn Screening (2010) issued by the Ministry of Health of the People’s Republic of China (Yang et al. 2017). In addition, a written informed consent on CH screening was obtained from the neonates’ parents prior to the collection of blood samples.

### Covariates

Considering that temperature was correlated with air pollution and the temperature has an impact on adverse birth outcomes (Baak et al. 2019), some studies also showed that toxic metals could affect the secretion of thyroid hormones in certain conditions (Nie et al. 2017), so the temperature and toxic metal in wastewater were taken as covariates. The temperatures in 30 provinces from January 1, 2014, to December 30, 2014, were also obtained from the Chinese Air Quality Online Monitoring and Analysis Platform similar to air pollution level calculation. The toxic metal data in wastewater contemporary with temperature were acquired from China Statistical Yearbook (2014) on the Chinese National Bureau of Statistics (National Bureau of Statistics of China 2015).

### Statistical analysis

The annual average exposure level of gaseous air pollution including SO_2_, NO_2_, O_3_, and CO in each province across China was calculated according to the monthly average level by the weighted average method. All the variables were conducted to normality test. In the descriptive analysis, data were presented as mean ± SD and percentile, but data that were not normally distributed were presented as only percentile. Then, Pearson correlation analysis and Spearman correlation analysis were used to analyze the potential correlation among variables.

The main aim and interest were to explore the association between maternal exposure level of gaseous air pollution and the incidence of newborn CH. Linear regression models were built for them. We fit the models with only gaseous air pollution and the incidence of newborn CH firstly; we estimated the changes in the incidence of newborn CH for an increase of 1 μg/m^3^ in SO_2_, NO_2_, and O_3_ and 1 mg/m^3^ in CO respectively in the single model; and we calculated the odd ratios (OR) and 95% confidence interval (95% CI). Then, considering the possible impacts of temperature and toxic metal, we adjusted for covariates including temperature (T), plumbum (Pb), hydrargyrum (Hg), arsenic (As), and cadmium (Cd) from wastewater in different models.

In the further analysis, maternal exposure to SO_2_, NO_2_, O_3_, and CO was categorized into quartiles (the 1st, 2nd, 3rd, and 4th quartiles of air pollution) based on the distribution according to their 25th, 50th, and 75th percentage. Multivariate logistic regression was built to examine the link between newborn CH risk and maternal gaseous air pollution exposure without or with adjustments of the covariates as well as in linear regression models. Based on the results from multivariate logistic regression, we determined the discriminatory property of a certain value of air pollution by constructing a receiver operator characteristic (ROC) curve and calculating the area under the curve (AUC). The ROC curve was applied to estimate the accuracy of analysis results and calculate cutoff values of air pollution, which may have the potential impact on the incidence of newborn CH according to the Youden index in the ROC curve.

We used SPSS Version 18.0 for all statistical analyses and a two-sided *p* value < 0.05 was considered statistically significant.

## Results

Data from 30 provinces in China were applied to the analysis, including the incidence of newborn CH from October 1, 2014, to September 30, 2015, gaseous air pollutants (SO_2_, NO_2_, CO, and O_3_) in 2014, toxic metal in wastewater and temperature in 2014 (January 1, 2014, to December 30, 2014). The incidence of CH and exposure level of air pollution were presented as mean ± SD. The average incidence of CH was 4.049±1.570 per 10,000 live birth; the average exposure levels of SO_2_, NO_2_, CO, and O_3_ were 31.329±14.481 μg/m^3^, 35.264±8.420 μg/m^3^, 1.176±0.291 mg/m^3^, and 80.462±13.127 μg/m^3^, respectively; and the average temperature was 29.307±5.800°C (Table 1). In addition, the toxic metals in wastewater were not normally distributed (Supplement table 1), so Pb, Hg, As, and Cd were described as the percentiles (Table 1).

**Table 1:** Summary statistics of CH, gaseous air pollutants, temperature, and toxic metal in wastewater in 30 provinces of China

Indicators^a^	Mean	SD	P25	P50	P75
CH	4.049	1.570	2.818	3.615	5.038
SO_2_	31.329	14.481	21.292	28.818	42.096
NO_2_	35.264	8.420	29.260	35.225	39.041
CO	1.176	0.291	0.988	1.174	1.325
O_3_	80.462	13.127	69.729	81.931	90.367
T	29.307	5.800	10.240	16.058	18.304
Pb	-	-	129.150	784.650	3890.950
Hg	-	-	5.025	10.550	30.575
As	-	-	70.825	872.800	3387.625
Cd	-	-	12.600	171.150	794.250

*SD* standard difference, *T* temperature, *P25* 25th percentile of the toxic metal according to distribution, *P50* 50th percentile of the toxic metal according to distribution, *P75* 75th percentile of the toxic metal according to distribution

^a^The unit of measurement for each indicator: 1 per 10,000 live birth for CH; μg/m^3^ for SO_2_, NO_2_, and O_3_; mg/m^3^ for CO; million tons for toxic metal in wastewater

We analyzed potential predictors of CH about gaseous air pollution, as well as the potential correlation among the four kinds of gaseous air pollutants with toxic metal in wastewater and temperature (Table 2). The incidence of newborn CH was positively correlated with O_3_ (*r*=0.435, *p*=0.016). Among the gaseous air pollutants, SO_2_ was positively correlated with NO_2_ and CO (*r*=0.406 and 0.619, respectively, both *p*<0.01) and NO_2_ positively correlated with CO and O_3_ (*r*=0.465 and 0.473, respectively, both *p*<0.01). Then, the coefficients were also as presented in Table 2 between CH and temperature, CH and toxic metal, and temperature and toxic metal.

**Table 2:** Correlation coefficient among various indicators in 30 provinces of China

Indicators	CH^a^	SO_2_^a^	NO_2_^a^	CO^a^	O_3_^a^	T^a^	Pb^b^	Hg^b^	As^b^	Cd^b^
CH^a^	1.000	0.107	0.307	−0.068	0.435*	0.233	0.156	−0.084	0.058	0.148
SO_2_^a^		1.000	0.406*	0.619^#^	0.144	−0.534^#^	−0.025	−0.009	−0.047	0.005
NO_2_^a^			1.000	0.465^#^	0.473^#^	−0.368^#^	−0.337	−0.333	−0.403*	−0.323
CO^a^				1.000	0.001	−0.345	0.157	0.251	0.085	0.143
O_3_^a^					1.000	−0.028	−0.034	−0.124	−0.139	−0.012
T^a^						1.000	0.301	0.181	0.200	0.273
Pb^b^							1.000	0.803^#^	0.924^#^	0.916^#^
Hg^b^								1.000	0.805#	0.834^#^
As^b^									1.000	0.913^#^
Cd^b^										1.000

*p<0.05; ^#^ *p*<0.01

^a^Variables obey normal distribution, the correlation coefficient according to Pearson bivariate analysis

^b^Variables do not obey normal distribution, the correlation coefficient between b and a or b according to Spearman bivariate analysis

The linear regression model showed that the incidence of newborn CH increased with the higher level of NO_2_ and O_3_ (Supplemental table 2). The newborn incidence of CH was increased under the maternal exposure to O_3_ level (Table 3), which was associated with an odds ratio of 1.053 (95% CI 1.013, 1.095) for a 1-μg /m^3^ increase in O_3_, observed in the unadjusted liner regression model (model 1). After adjusting for temperature, toxic metal, temperature, and toxic metal in different models, an increase in the incidence of newborn CH for a 1-μg/m^3^ increase in O_3_ level was also observed, which was associated with an odds ratio of 1.054 (95% CI 1.014, 1.097), 1.052 (95% CI 1.008, 1.099), and 1.055 (95% CI 1.011, 1.102), respectively. For maternal exposure to NO_2_, we found that maternal exposure to NO_2_ per 1-μg/m^3^ increase was positively associated with the risk of newborn CH adjusted for temperature (OR 1.089, 95% CI 1.019, 1.164) and adjusted for temperature and toxic metal (OR 1.097, 95% CI 1.019, 1.182).

**Table 3:** The OR (95% CI) of SO_2_, NO_2_, CO, and O_3_ exposure and the incidence of newborn CH in different models by liner regression analysis

Air pollutants	OR (95% CI)	*P*
SO_2_		
Model 1	0.988 (0.950,1.028)	0.574
Model 2	1.003 (0.957,1.051)	0.900
Model 3	0.992 (0.950,1.036)	0.728
Model 4	1.001 (0.951,1.053)	0.976
NO_2_		
Model 1	1.059 (0.990,1.132)	0.098
Model 2	*1.089 (1.019,1.164)*	*0.018*
Model 3	1.077 (1.000,1.182)	0.058
Model 4	*1.097 (1.019,1.182)*	*0.022*
CO		
Model 1	0.694 (0. 094,5.100)	0.722
Model 2	1.081 (0.131,8.908)	0.943
Model 3	0.816 (0.094,7.105)	0.855
Model 4	1.073 (0.107,10.793)	0.953
O_3_		
Model 1	*1.053 (1.013,1.095)*	*0.016*
Model 2	*1.054 (1.014,1.097)*	*0.013*
Model 3	*1.052 (1.008,1.099)*	*0.029*
Model 4	*1.055 (1.011,1.102)*	*0.024*

Model 1, unadjusted for covariates in linear regression. Model 2, adjusted for the average temperature in linear regression. Model 3, adjusted for the content of toxic metal in wastewater, including Pb, Hg, As, and Cd in linear regression. Model 4, adjusted for the average temperature and the content of toxic metal in wastewater including Pb, Hg, As, and Cd in linear regression

**Fig. 1:**
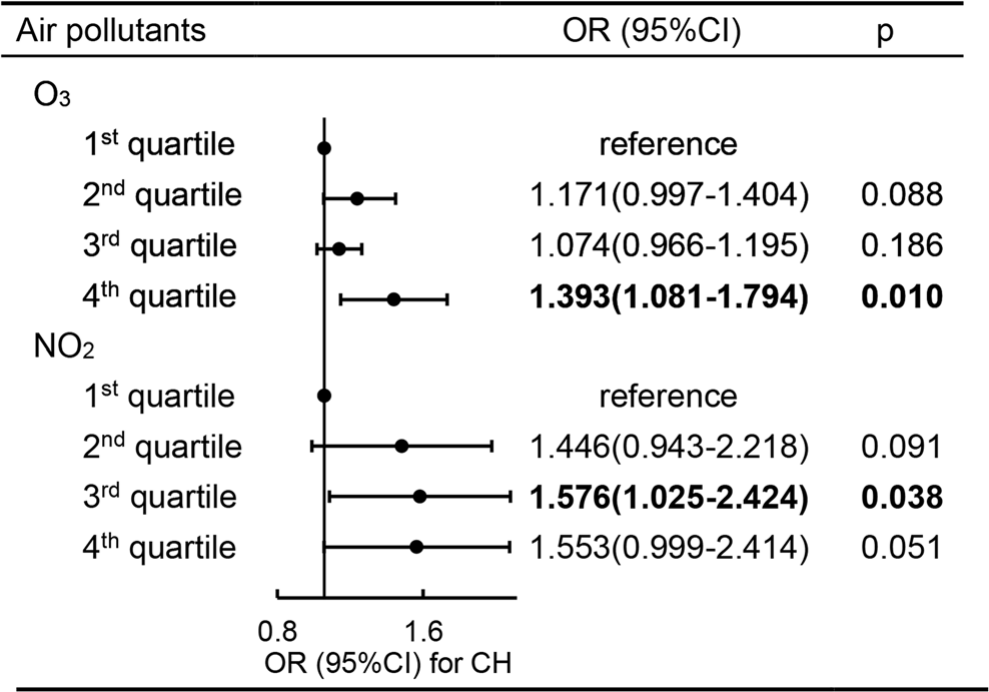
The OR (95% CI) of different levels of maternal exposure to O_3_ and NO_2_ and the newborn incidence of CH after being adjusted for temperature and toxic metal in wastewater in multivariate logistic regression

In logistic regression analysis (Fig. 1), the incidence of CH in newborns was categorized into four categories according to the percentile (the 25th, 50th, and 75th percentiles of air pollutants). Compared with the lowest level of O_3_ (the 1st quartile of O_3_), maternal exposure to the highest level of O_3_ (the 4th quartile of O_3_) was associated with the increased incidence of CH, was associated with an odds ratio of 1.393 (95% CI 1.081–1.794) after adjusting for temperature and toxic metal in wastewater. For NO_2_, the newborn incidence of CH was also increased significantly under maternal exposure to the 3rd quartile of NO_2_ and, after adjusting for temperature and toxic metal in wastewater, was associated with an odds ratio of 1.576 (95% CI 1.025–2.424) compared with the lowest level maternal exposure to NO_2_, as well as the maternal exposure to the highest level of NO_2_ (OR 1.553, 95% CI 0.999–2.414).

**Fig. 2:**
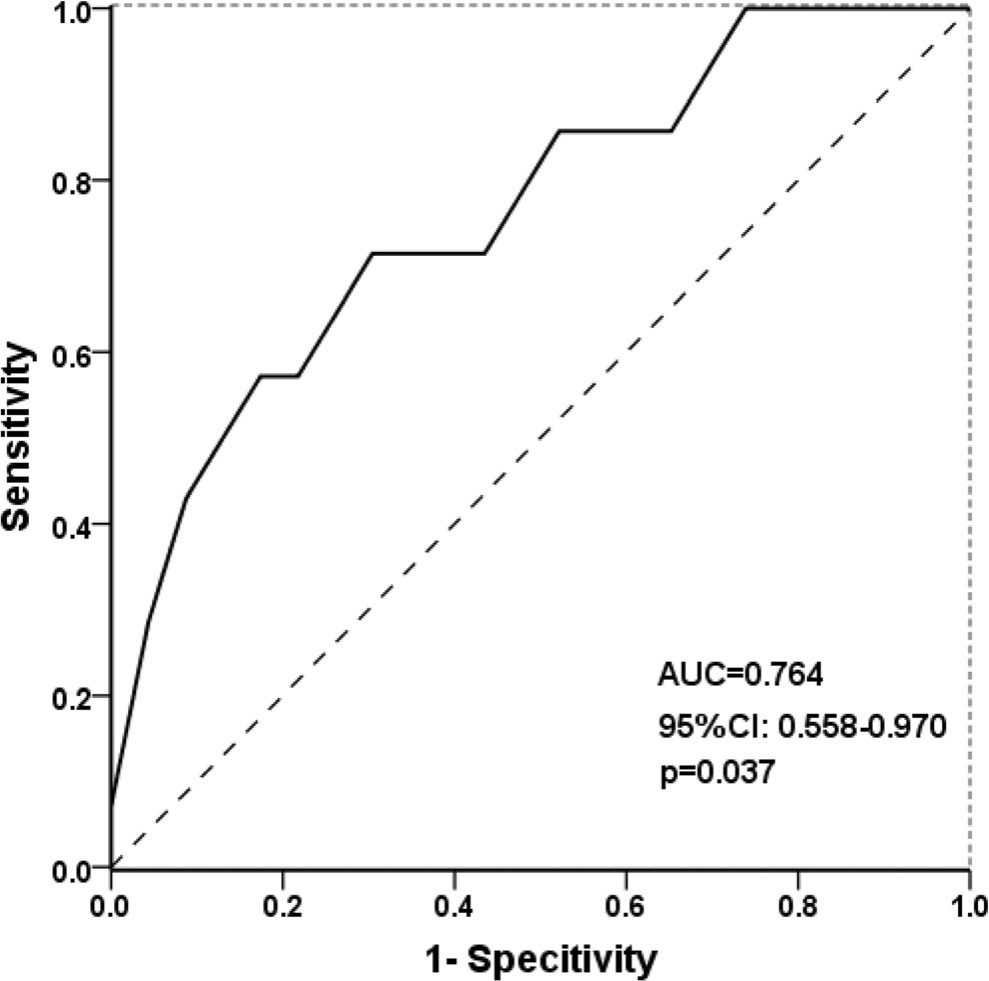
The ROC curve of O_3_ for predicting the risk of newborn CH

Furthermore, we categorized O_3_ and NO_2_ in two categories (below and above the 75th percentile for O_3_ and the 50th percentile for NO_2_); according to the multivariate logistic regression, the ROC curve was constructed to estimate the accuracy of the analysis and the ability of O_3_ and NO_2_ to predict the risk of newborn CH (Fig. 2). For the maternal exposure to O_3_, the AUC (the area between the curve and the reference line) in the ROC curve was more than 0.500 (AUC=0.764, 95% CI: 0.558, 0.970, *p*=0.037), and when the concentration of O_3_ was 93.688 μg /m^3^, the Youden index was biggest in the ROC curve (Supplemental table 3). But the ROC curve about NO_2_ had no significance. (the data was not shown).

## Discussion

In the data-based analysis in 30 provinces of China, we found that maternal exposure to O_3_ and NO_2_ during pregnancy was positively associated with the risk of newborn CH, which revealed that O_3_ and NO_2_ might be the risk factor in the occurrence of newborn CH. As we know, the hypothyroidism during infancy and childhood will have a great impact on the physical growth and mental development in the future especially in nerve and brain development by the hypothalamic–pituitary–thyroid axis (Shields et al. 2011). The increased risk of the incidence of newborn CH was observed with the higher level of air pollution by linear regression analysis and multivariate logistic regression. In linear regression analysis, per 1-μg/m^3^ increase of O_3_ and NO_2_ associated with a higher risk of newborn CH (adjusted OR 1.055, 95% CI 1.011, 1.102 for O_3_ and adjusted OR 1.097, 95% CI 1.019, 1.182 for NO_2_, after adjusting for temperature and toxic metal in wastewater). Compared with the lowest level of air pollution, the highest level of O_3_ positively associated with newborn CH (adjusted OR 1.393, 95% CI 1.081, 1.794), and maternal exposure to the 3rd quartile of NO_2_ also associated with higher risk of newborn CH (adjusted OR 1.576, 95% CI 1.025, 2.424). For the highest level of NO_2_ (adjusted OR 1.533 and 95% CI 0.999–2.414), also could be considered significantly as the risk level for the occurrence of newborn CH. The 75th percentile of O_3_ as predictor to fit the ROC curve between the incidence of newborn CH and gaseous air pollution, the aim to find the potential cutoff of air pollutants that impacted the newborn CH in China. A rational cutoff value of O_3_ was calculated to be 93.688 μg/m^3^ located in the biggest Youden index (Supplemental table 3). This value is very close to the 75th percentile of O_3_ (90.367 μg/m^3^). So, controlling the O_3_ concentration below the level of 93.688 μg/m^3^ may decrease the risk of newborn CH. But, in the ROC curve, the significant association between NO_2_ and CH was not found when categorized according the 50th percentile as the most common value to distinguish the high or low level of CH. The potential reason might be that the ROC model cannot be adjusted for covariables. However, the AUC between CH and NO_2_ is very close to 0.500, and we also should pay attention to the 50th percentile of NO_2_ on the risk of newborn CH.

In this research, the association between maternal exposure to SO_2_ and CO and newborn CH was not observed. We cannot rule out the potential impacts of maternal exposure to the other air pollutants on the occurrence of newborn CH because of the failure to observe the associations in the present study. Previous studies identified that maternal exposure to SO_2_ and CO has a certain impact on adverse birth outcomes (Lamichhane et al. 2018; Robledo et al. 2015). Perhaps the effects will be found through the expansion of sample size. The further studies should be conducted.

The current studies showed that maternal exposure to air pollution during pregnancy affected the level of thyroid hormones and TSH, as well as newborn thyroid function (Dallaire et al. 2009; Howe et al. 2018; Janssen et al. 2017). Some adverse birth outcomes were closely related to thyroid dysfunction under certain conditions (Herr et al. 2010). Studies focused on association between maternal exposure to air pollution and newborn CH are relatively limited; therefore, we conducted this analysis. On air pollution, people paid more attention to PM, but the gaseous air pollution also important. China is a country with huge traffic burden. Air pollution has become a major social issue, especially NO_2_, SO_2_, CO, and O_3_ which can be transformed to photochemical smog under the role of sunlight, which had more serious harm on women and fetuses. It is easier for gaseous air pollution to enter the respiration tract and to be exchanged into blood through the lungs. The newborn CH will have a lasting effect on the human body, and the incidence of newborn CH (4.1 per 10,000 live births) is higher than the level worldwide (Deng et al. 2018); it is essential to pay attention to the incidence of CH.

Numerous studies have confirmed that maternal exposure to air pollution is positively associated with the risk of maternal diseases (Robledo et al. 2015) (Nachman et al. 2016; Pedersen et al. 2014) and fetal diseases such as PTB, SGA, LBW, and birth defects (Geer et al. 2012; Girguis et al. 2016; Gray et al. 2014; Lamichhane et al. 2018). These diseases may share the same mechanism or pathogenesis with CH. Maternal exposure to air pollution influences the expression of placental genes and the cellular signaling pathway (Saenen et al. 2015); people lived in bad environments with higher level of DNA adducts in the blood and increased probability of genetic mutation (Tang et al. 2014). Fetal lymphocyte ratio (T/B cell) in early pregnancy (Herr et al. 2010) and the levels of thyroid-related hormones (Janssen et al. 2017) are also affected by maternal exposure to air pollution. In addition, studies found that maternal exposure to air pollution is associated with inflammation and oxidative stress in pregnant women, and even influenced the function and blood perfusion of plasma (Chuang et al. 2007; Lane et al. 2016; Schembari et al. 2014); all the changes may affect the occurrence of CH in the offspring. These studies provide us an idea to explore the potential influence of maternal exposure to air pollution on the occurrence of newborn CH. But the mechanism was still indefinite and further studies should be performed.

Multivariate logistic regression was performed, and the accuracy in the ROC curve between maternal exposure to O_3_ and the incidence of newborn CH in the newborn was estimated. The concentration of 93.688 μg/m^3^ in O_3_ is significant in predicting the risk of newborn CH. The ROC curve is the most common way to calculate the cutoff. So, the 93.688 μg/m^3^ in O_3_ might be served as the cutoff to predict the risk of CH. The World Health Organization (WHO) Air quality guidelines (https://www.who.int/) set the guideline value for the O_3_ level at 100 μg/m^3^ for an 8-h daily average value (the 8-h daily average value referred to the highest cumulative average level based on the consecutive 8 h of O_3_ concentration from 8:00 am to 12:00 pm of the day). The cutoff 93.688 μg/m^3^ in O_3_ is very close to the guideline value (100 μg/m^3^) and belongs to the high level of O_3_ (categorized according to the 75th percentile of O_3_), so to some extent the value has the predicted significance, according to the WHO Air quality guidelines. There were still some provinces where the level of air pollution exceeded the guideline value; the air contamination in China should be controlled continuously. Although the study was based on air pollution in China, the cutoff value might be applied to other regions. It is significant for China as a populous country. At last, the cutoff value of O_3_ in the present study was 93.688 μg/m^3^, below which the risk of newborn CH could be effectively reduced.

It was difficult to avoid some limitations in the present study. We were unable to obtain the general demographic characteristics from the national databases. And the present study did not adjust more confounding factors including parity, maternal residence, maternal gestational age, family income status, and family history of thyroid diseases, which eventually influenced the analysis results and caused a certain bias. The main advantage of the present study was the combination of two national databases, where the data covered the most of provinces in China. The other advantage was that the data were verified by professionals in the databases, which was helpful to minimize the bias caused by investigators and respondents, avoid the misclassification effectively in the procession of data collection. In addition, the calculating of cutoff value of O_3_ provided a potential epidemiological evidence for the establishment of an interim target to control air pollution in China, which may be beneficial to reduce the occurrence of CH and safeguard the health in the offspring.

In conclusion, maternal exposure to O_3_ and NO_2_ during pregnancy may influence the occurrence of newborn CH. Meanwhile, we are supposed to alert the impacts from maternal exposure to SO_2_ and CO on newborn CH. We would better to maintain the O_3_ level under 93.688 μg/m^3^ which may be beneficial to control the risk of CH and safeguard health in the offspring in China.

## Supplementary information

ESM 1 (DOCX 15 kb)

ESM 2 (DOCX 16 kb)

ESM 3 (DOCX 17 kb)

## Data Availability

The data generated and used in the analysis of the present study are included in published article. Additional data is available from the authors upon reasonable request.
