# Supplementary material for: Maternal exposure to O3 and NO2 may increase the risk of newborn congenital hypothyroidism: a national data-based analysis in China
Source: Environ Sci Pollut Res Int. 2021 Mar 2;28(26):34621–9. doi: 10.1007/s11356-021-13083-6 (PMC8275538; doi:10.1007/s11356-021-13083-6)
Supplement: Supplementary file 1 — (DOCX 15 kb) [file 11356_2021_13083_MOESM1_ESM.docx]

**Supplemental table 1**

The test of normally distribution for variables.

| Indicator | Kolmogorov-Smirnov(K) | | Shapiro-Wilk | |
| --- | --- | --- | --- | --- |
|  | Statistical value | P | Statistical value | P |
| CH | 0.144 | 0.116 | 0.936 | 0.070 |
| SO2 | 0.127 | 0.200 | 0.966 | 0.442 |
| NO2 | 0.146 | 0.102 | 0.958 | 0.268 |
| CO | 0.095 | 0.200 | 0.979 | 0.805 |
| O3 | 0.083 | 0.200 | 0.978 | 0.775 |
| Temperature | 0.096 | 0.200 | 0.082 | 0.880 |
| Pb | 0.282 | ＜0.05 | 0.583 | ＜0.05 |
| Hg | 0.271 | ＜0.05 | 0.689 | ＜0.05 |
| As | 0.314 | ＜0.05 | 0.526 | ＜0.05 |
| Cd | 0.318 | ＜0.05 | 0.467 | ＜0.05 |

The *p*<0.05 meant the data distribution normally.
