# Supplementary material for: Maternal exposure to O3 and NO2 may increase the risk of newborn congenital hypothyroidism: a national data-based analysis in China
Source: Environ Sci Pollut Res Int. 2021 Mar 2;28(26):34621–9. doi: 10.1007/s11356-021-13083-6 (PMC8275538; doi:10.1007/s11356-021-13083-6)
Supplement: Supplementary file 2 — (DOCX 16 kb) [file 11356_2021_13083_MOESM2_ESM.docx]

**Supplemental table 2**

The B-value for the newborn incidence of CH and air pollution in linear regression.

| Air pollutants |  |  |  | | The incidence of newborn CH | | | | | |
| --- | --- | --- | --- | --- | --- | --- | --- | --- | --- | --- |
|  | B^a^ | | P^a^ | B^b^ | | P^b^ | B^c^ | P^c^ | B^d^ | P^d^ |
| SO_2_ | 0.018 | | 0.574 | 0.003 | | 0.900 | -0.008 | 0.078 | 0.001 | 0.967 |
| NO_2_ | 0.057 | | 0.098 | **0.109** | | **0.036** | 0.074 | 0.058 | **0.093** | **0.022** |
| CO | -0.366 | | 0.722 | 0.078 | | 0.242 | -0.203 | 0.855 | 0.070 | 0.953 |
| **O_3_** | **0.052** | | **0.016** | **0.053** | | **0.013** | **0.051** | **0.029** | **0.054** | **0.024** |

B for the incidence change of newborn CH associated with a 1μg/m^3^ increase in SO_2_, NO_2_, O_3_ and 1mg/m^3^ in CO.

^a^ unadjusted for covariates in linear regression.

^b^ adjusted for the average temperature in linear regression.

^c^ adjusted for the content of toxic metal in wastewater, including Pb, Hg, As and Cd in linear regression.

^d^ adjusted for the average temperature and the content of toxic metal in wastewater including Pb, Hg, As and Cd in linear regression
