# Supplementary material for: Maternal exposure to O3 and NO2 may increase the risk of newborn congenital hypothyroidism: a national data-based analysis in China
Source: Environ Sci Pollut Res Int. 2021 Mar 2;28(26):34621–9. doi: 10.1007/s11356-021-13083-6 (PMC8275538; doi:10.1007/s11356-021-13083-6)
Supplement: Supplementary file 3 — (DOCX 17 kb) [file 11356_2021_13083_MOESM3_ESM.docx]

**Supplemental table 3**

Sensitivity, specificity and Youden index changes at different O_3_ exposure concentrations

| O_3_(ug/m^3^) | Sensitivity | specificity | Yordan index |
| --- | --- | --- | --- |
| **93.688** | **0.571** | **0.957** | **1.528** |
| 91.061 | 0.571 | 0.913 | 1.484 |
| 81.931 | 0.857 | 0.609 | 1.466 |
| 90.44 | 0.571 | 0.870 | 1.441 |
| 80.722 | 0.857 | 0.565 | 1.422 |
| 89.663 | 0.571 | 0.826 | 1.398 |
| 97.461 | 0.429 | 0.957 | 1.385 |
| 79.177 | 0.857 | 0.522 | 1.379 |
| 83.847 | 0.714 | 0.652 | 1.366 |
| 88.640 | 0.571 | 0.783 | 1.354 |
| 70.500 | 1.000 | 0.348 | 1.348 |
| 76.885 | 0.857 | 0.478 | 1.335 |
| 82.833 | 0.714 | 0.609 | 1.323 |
| 87.354 | 0.571 | 0.739 | 1.311 |
| 69.125 | 1.000 | 0.304 | 1.304 |
| 74.785 | 0.857 | 0.435 | 1.292 |
| 99.578 | 0.286 | 1.000 | 1.286 |
| 85.783 | 0.571 | 0.696 | 1.267 |
| 67.438 | 1.000 | 0.261 | 1.261 |
| 72.717 | 0.857 | 0.391 | 1.248 |
| 99.125 | 0.286 | 0.957 | 1.242 |
| 84.943 | 0.571 | 0.652 | 1.224 |
| 66.888 | 1.000 | 0.217 | 1.217 |
| 71.141 | 0.857 | 0.348 | 1.205 |
| 65.867 | 1.000 | 0.174 | 1.174 |
| 101.859 | 0.143 | 1.000 | 1.143 |
| 64.500 | 1.000 | 0.13 | 1.13 |
| 61.458 | 1.000 | 0.087 | 1.087 |
| 56.000 | 1.000 | 0.043 | 1.043 |
| 52.167 | 1.000 | 0.000 | 1.000 |
